# Prmt5 is a regulator of muscle stem cell expansion in adult mice

**DOI:** 10.1038/ncomms8140

**Published:** 2015-06-01

**Authors:** Ting Zhang, Stefan Günther, Mario Looso, Carsten Künne, Marcus Krüger, Johnny Kim, Yonggang Zhou, Thomas Braun

**Affiliations:** 1 Department of Cardiac Development and Remodeling, Max-Planck Institute for Heart and Lung Research, Bad Nauheim 61231, Germany

## Abstract

Skeletal muscle stem cells (MuSC), also called satellite cells, are indispensable for maintenance and regeneration of adult skeletal muscles. Yet, a comprehensive picture of the regulatory events controlling the fate of MuSC is missing. Here, we determine the proteome of MuSC to design a loss-of-function screen, and identify 120 genes important for MuSC function including the arginine methyltransferase Prmt5. MuSC-specific inactivation of *Prmt5* in adult mice prevents expansion of MuSC, abolishes long-term MuSC maintenance and abrogates skeletal muscle regeneration. Interestingly, Prmt5 is dispensable for proliferation and differentiation of Pax7^+^ myogenic progenitor cells during mouse embryonic development, indicating significant differences between embryonic and adult myogenesis. Mechanistic studies reveal that Prmt5 controls proliferation of adult MuSC by direct epigenetic silencing of the cell cycle inhibitor *p21*. We reason that Prmt5 generates a poised state that keeps MuSC in a standby mode, thus allowing rapid MuSC amplification under disease conditions.

## Abstract (web summary)

Skeletal muscle satellite cells are important for muscle regeneration, but their regulatory mechanisms are largely unknown. Here the authors identify arginine methyltransferase Prmt5 as a key regulator of satellite cell maintenance and function in adult mice, and show that Prmt5 acts mainly but not exclusively on the cell cycle inhibitor p21.

## Graphical abstract

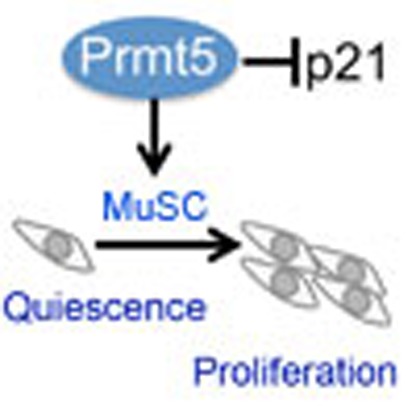

Organ-specific adult stem cells enable continuous regeneration of various tissues throughout adult life. Most adult stem cells are assumed to undergo constant turnover to ascertain self-renewal and tissue homeostasis, although dormant adult stem cells have been described in the haematopoietic system, which remain in a quiescent state for most of their lifetime and divide only rarely under severe stress conditions^1^,^2^. Adult skeletal muscle stem cells (MuSC) are represented by a specialized subset of myofibre-associated cells called satellite cells and own a remarkable regenerative potential, which enables them to continuously replace myofibres by undergoing repeated rounds of activation and expansion under persisting disease conditions. Satellite cells originate during early embryonic development from a population of proliferating cells of the paraxial mesoderm. While most cells activate myogenic genes and form skeletal muscle fibres, some remain undifferentiated, adopt a satellite cell position in postnatal muscle and acquire a quiescent state^3^,^4^,^5^. After injury or excessive exercise, Pax7^+^ cells exit quiescence, proliferate and differentiate to generate new myofibres or fuse with existing myofibres, thereby fully restoring damaged skeletal muscle tissues^6^,^7^,^8^,^9^. Several recent studies demonstrated that ablation of Pax7^+^ MuSCs prevents muscle repair under pathological conditions and during ageing (review in ref. 10). However, the molecular mechanisms that control satellite cell functions during skeletal muscle regeneration are only partially understood, although several factors directing the fate of MuSC have been identified (reviewed in refs 2, 11).

To identify new regulators of MuSC activation, self-renewal, expansion and differentiation, we establish a systematic screening approach taking advantage of mass spectrometry-based protein profiling of FACS (fluorescence-activated cell sorting)-sorted MuSC^6^,^12^,^13^ combined with short hairpin RNA (shRNA)-mediated knockdown of MuSC-specific genes. Several genes are identified that strongly affect activities of MuSC *in vitro*, of which the histone arginine methyltransferase Prmt5 is chosen for in-depth functional analysis. We find that Prmt5 is essential for adult MuSC proliferation and muscle regeneration by restricting *p21* expression via direct epigenetic silencing, thereby allowing rapid expansion of MuSC. Since the lack of Prmt5 does not affect embryonic myogenesis, we postulate that prenatal muscle development and adult muscle regeneration use distinct genetic and epigenetic mechanisms for the control of muscle progenitor cell expansion.

## Results

### Identification of novel regulators controlling MuSC homeostasis

**Figure 1:**
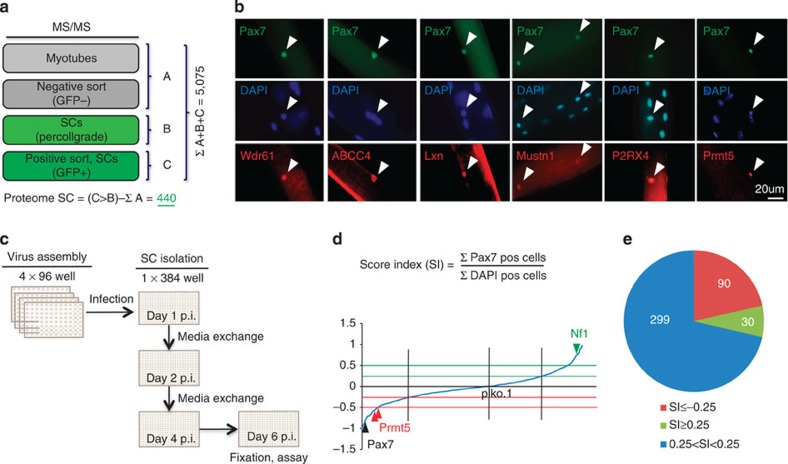
Identification of regulators of stem cell homeostasis in a MuSC proteome-based shRNA screen. (**a**) Mass spectrometry (MS)-based identification of MuSC-enriched proteins (Proteome SC) using samples from fractionated skeletal muscles including purified myotubes, Pax7-GFP^−^ mononuclear cells, percoll-gradient purified and Pax7-GFP^+^ MuSC. (**b**) Immunofluorescence validation of identified proteins (red) counterstained with Pax7 antibody (green) and DAPI (blue; scale bar, 20 μm). (**c**) Schematic outline of the shRNA screen against corresponding genes of the satellite cell proteome. Phenotypic scores are calculated as ratios of Pax7^+^/DAPI^+^ nuclei for each well. (**d**) Poisson distribution of relative Pax7 expression for all 2,226 shRNAs targeting 419 genes. Red and green lines indicate phenotypic scores lower or higher than 0.25 percentiles normalized to plko.1 empty vector control. Knockdown of Pax7 reduces proliferation and enhances differentiation of MuSC^6^, whereas knockdown of Nf1 increases the numbers of Pax7 expressing cells^51^. (**e**) shRNA knockdown identifies 90 and 30 candidate genes causing down- and upregulation of Pax7^+^/DAPI^+^ ratios, respectively. P.i., post infection; Pos, positive.

To determine the proteome of MuSCs, we isolated GFP-labelled stem cells (SC^GFP^) from skeletal muscles of *Pax7*^*ICN*^*/ZEG* mice^6^,^14^ via FACS (Supplementary Fig. 1a), which all expressed Pax7 protein and readily differentiated into myocytes (Supplementary Fig. 1b,c). Protein extracts of freshly isolated MuSCs were subjected to mass spectrometry analysis (*n*=3) resulting in the identification of 135,341 peptides in all samples combined corresponding to 5,031 proteins in MuSC with at least one unique peptide (Supplementary Data 1). Notably, we detected numerous proteins known to be highly expressed in MuSCs including CD34, integrin α7, caveolin-1, Numb and β1-integrin, but not haematopoietic or endothelial cell markers such as CD45 or CD31 (refs 12, 13, 15). Comparison with proteome data sets obtained from myofibres, Pax7^-^ mononuclear cells and the MuSC^PG^ fraction (satellite cells after percoll gradient and before FACS sorting) allowed us to identify 441 proteins that are exclusively present in MuSC but not in differentiated myofibres (Fig. 1a and Supplementary Data 2). Mass spectrometry data of randomly selected proteins (Wdr61, ABCC4, Lxn, Mustn1, P2RX4 and Prmt5) were validated by immunofluorescence staining of freshly isolated myofibres (Fig. 1b).

To analyse the function of MuSC-specific proteins, we generated a custom-arrayed lentiviral shRNA library (400 genes, one shRNA per well, on average five different shRNAs per gene; Fig. 1c and Supplementary Data 2). FACS-purified *Pax7*^*ZsGreen*^ MuSC^12^ were transduced with shRNA expressing lentiviruses and analysed by high-throughput fluorescent microscopy 96 h post transduction for the ratio of Pax7^+^ versus total 4,6-diamidino-2-phenylindole (DAPI)^+^ cells (Fig. 1d), providing a read-out for genes affecting self-renewal, proliferation and differentiation of MuSC. shRNAs targeting Pax7 and Nf1 were included as quality controls (*n*=4 wells for each plate). After selecting for genes, which yielded a strong shift of the ratio of Pax7^+^ versus total DAPI^+^ cells after knockdown, we ended up with 30 genes inducing and 90 genes decreasing Pax7/DAPI^+^ cell ratios after knockdown (Fig. 1e, Supplementary Fig. 1d and Supplementary Data 3).

### *Prmt5* is required for muscle regeneration

**Figure 2:**
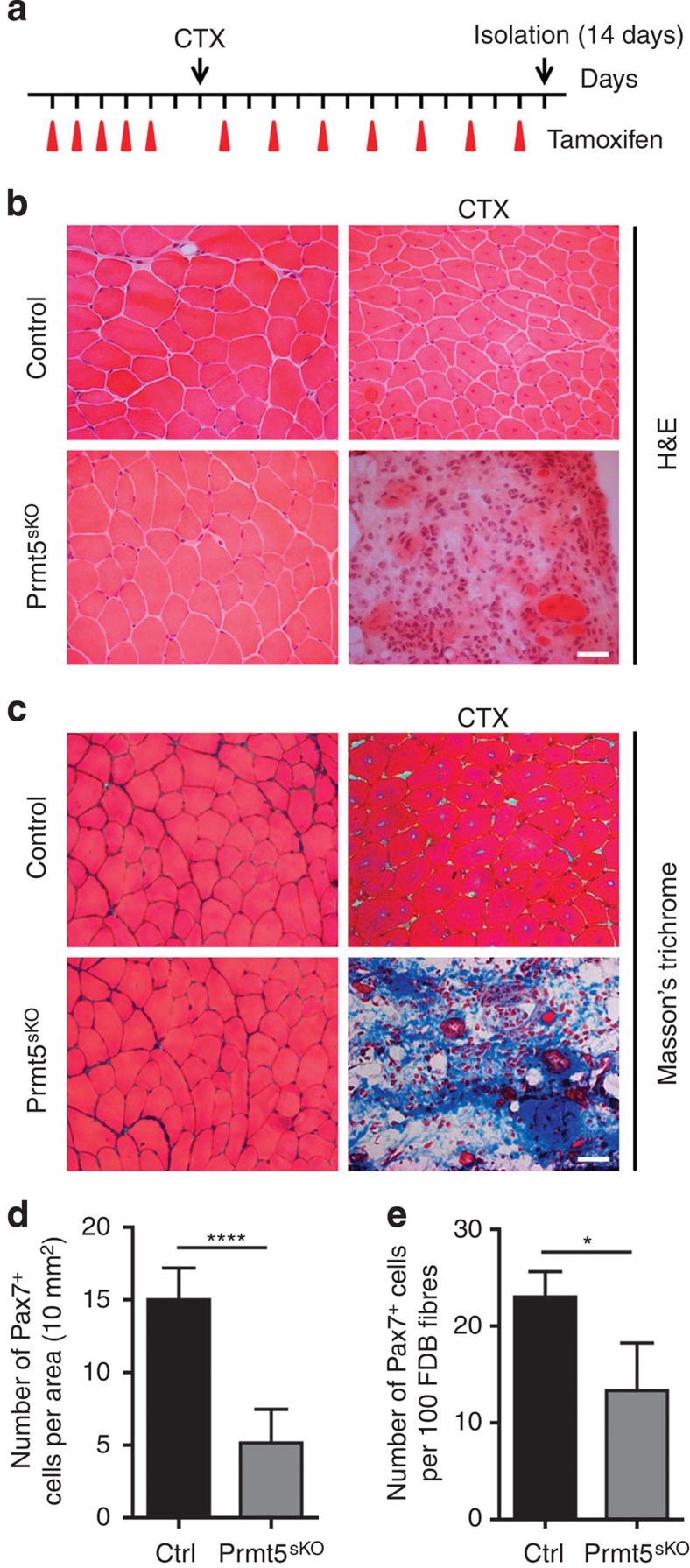
Prmt5 is required for muscle regeneration and long-term maintenance of MuSC. (**a**) Schematic outline of CTX injection in TAM-treated control and *Prmt5*^*sKO*^ mutant littermates at the age of 8–9 weeks. (**b**) Haematoxylin and eosin (H&E) and (**c**) Masson's Trichrome staining of injured or uninjured TA muscles of *Prmt5*^*sKO*^ mice and control littermates (*n*=3, each) 14 days after CTX injection. Scale bar, 20 μm. (**d**,**e**) Number of Pax7^+^ cells 4 months after TAM administration on TA cryosections (*n*=6, each; **d)** and freshly isolated FDB myofibres (*n*=3, each; **e**) of *Prmt5*^*sKO*^ and control mice. The number of Pax7^+^ cell per 10 mm^2^ (**d**) or per 100 fibres (**e**) was counted. Error bars represent s.d.'s of the mean (*t*-test: ****P<0.0001, *P<0.05; *n*=3). Control (Ctrl): *Pax7CreERT2*^+/−^*/Prmt5*^*+/loxP*^; *Prm5*^*sKO*^: *Pax7CreERT2*^+/−^*/Prmt5*^*loxP/loxP*^.

Next, we initiated a thorough analysis of the function of an exemplary candidate, the arginine methyltransferase Prmt5, that mediates H3R8 symmetric dimethylation (H3R8me2s; ref. 16). Prmt5 was recently implicated in the regulation of proliferation of embryonic stem cells^17^,^18^ and neural progenitor cells (NPCs) during brain development^19^, but its function in adult stem cells has remained elusive. Inactivation of *Prmt5* using *Prmt5*^*loxP/loxP*^ (Supplementary Fig. 2a) and *Pax7*^*CreERT2*^ mice^6^,^7^ (=MuSC-specific Prmt5 knockout mice, *Prmt5*^*sKO*^) (Supplementary Fig. 2b) efficiently depleted Prmt5 mRNA in adult MuSC (Ctrl: 1.000±0.339, *n*=3; *Prmt5*^*sKO*^: 0.033±0.020, *n*=4; Supplementary Fig. 2c). Interestingly, tamoxifen (TAM)-treated *Prmt5*^*sKO*^ mice remained viable and displayed no obvious phenotype under physiological conditions 21 days after treatment compared with control animals (Ctrl=*Pax7*^*CreERT2*^*/Prmt5*^*+/loxP*^; Supplementary Fig. 2d). No significant change of body weight (Ctrl 23.60±3.18; *Prmt5*^*sKO*^ 22.67±2.52, each *n*=3) and no morphological alterations of skeletal muscle tissue were apparent (Supplementary Fig. 2e f). Likewise, the number of satellite cells on sections of tibialis anterior (TA) muscle (Ctrl 20.33±2.08; *Prmt5*^*sKO*^ 20.33±1.53, each *n*=3) and freshly isolated flexor digitorum brevis (FDB) myofibres (Ctrl 30.00±3.61; *Prmt5*^*sKO*^ 30.00±2.00, each *n*=3) did not differ between control and mutant littermates (Supplementary Fig. 2g,h). To investigate whether Prmt5-deficient satellite cells contribute to muscle regeneration, TA muscles of TAM-treated *Prmt5*^*sKO*^ and control littermates were injected with cardiotoxin (CTX; Fig. 2a and Supplementary Fig. 2i). Strikingly, muscle regeneration was completely abolished in *Prmt5*^*sKO*^ mice at all investigated time points (7 and 14 days, and 4 months after injury). The virtually complete lack of regenerated muscle fibres (Fig. 2b and Supplementary Fig. 2j) was accompanied by a massive increase of fibrosis (Fig. 2c and Supplementary Fig. 2k). To analyse whether Prmt5 affects long-term satellite cell maintenance, we determined the number of MuSC 4 months after the initial TAM treatment. Importantly, we detected a significant decline of Pax7^+^ MuSC numbers both on cryosections from TA muscles (Fig. 2d; Ctrl 15.00±2.19; *Prmt5*^*sKO*^ 5.17±2.32, each *n*=6) and on freshly isolated myofibres from FDB muscle (Ctrl 23.00±2.65; *Prmt5*^*sKO*^ 13.33±4.93, each *n*=3; Fig. 2e), indicating that Prmt5 is required for MuSC expansion during regeneration and needed to replenish the MuSC niche during physiological ageing.

### Prmt5 prevents depletion of the MuSC pool in *mdx* mice

**Figure 3:**
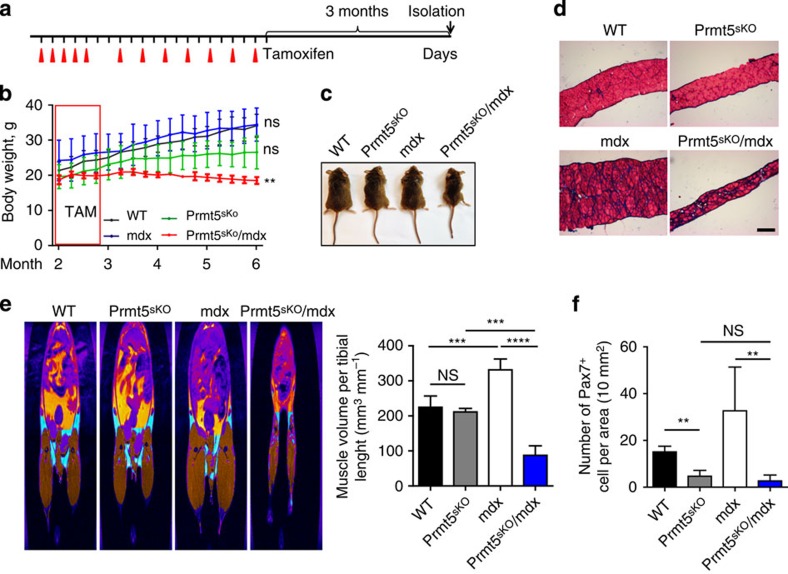
Continuous muscle regeneration depletes the MuSC pool of *Prmt5*^*sKO*^ mice. (**a**) Schematic outline of the TAM administration and analysis of wild-type (WT), *Prmt5*^*sKO*^, *mdx* and *Prmt5*^*sKO*^*/mdx* mice (*n*=3, each). (**b–d**) *Prmt5*^*sKO*^*/mdx* mice (*n*=3) show lower body weight (**b**,**c**) and thinner diaphragm (**d**) compared with WT (*n*=3), mdx (*n*=3) and *Prmt5*^*sKO*^ (*n*=3) littermates 3 months after TAM administration. Scale bar, 100 μm (**d**). (**e**) MRI measurements of decreased and increased muscle volume (brown colour) in *Prmt5*^*sKO*^*/mdx* and *mdx* mice, respectively, 3 months after TAM administration (WT and *mdx*, *n*=5 each; *Prmt5*^*sKO*^, *n*=3; *Prmt5*^*sKO*^*/mdx*, *n*=4). Quantification of muscle volume normalized to tibia length is shown on the right. Error bars represent s.d.'s of the mean (*t*-test: ****P<0.0001; ***P<0.001; NS, *P*>0.05). (**f**) Decreased numbers of Pax7^+^ cells in TA muscles of *Prmt5*^*sKO*^*/mdx* mice (*n*=5) compared with WT (*n*=5 and *mdx* (*n*=3) littermates. The number of Pax7^+^ cells per 10 mm^2^ area was counted on cryosections. Error bars represent s.d.'s of the mean (*t*-test: **P<0.01; NS, *P*>0.05). Control (Ctrl): *Pax7CreERT2*^+/−^*/Prmt5*^*+/loxP*^; *Prm5*^*sKO*^: *Pax7CreERT2*^+/−^*/Prmt5*^*loxP/loxP*^. NS, not significant.

To further explore the role of Prmt5 in replenishing the MuSC pool, we utilized *mdx* mice, which lack functional dystrophin resulting in continuous degeneration/regeneration of myofibres, accompanied by repeated activation and enhanced turnover of satellite cells^20^. Treatment of 8-week-old *Prmt5*^*sKO*^*/mdx* compound mutant mice for 3 weeks with TAM (Fig. 3a) resulted in progressive loss of body weight, whereas *Prmt5*-deficient and *mdx* mice gained weight similar to wild-type littermates (Fig. 3b,c). *Prmt5*^*sKO*^*/mdx* mice had a markedly lower body weight 4 months after initiation of the TAM treatment (Fig. 3b,c), and the diaphragm was markedly thinner compared with controls (Fig. 3d). Magnetic resonance imaging (MRI) measurements revealed a massive decrease of the total muscle mass normalized to tibia length of *Prmt5*^*sKO*^*/mdx* (87.5±27.2 mm^3^ per mm, *n*=4) but not of *Prmt5* mutants (211.5±10.0 mm^3^ per mm, *n*=3), wild-type mice (224.9±32.3 mm^3^ per mm, *n*=5) and *mdx* mice (332.0±30.8 mm^3^ per mm, *n*=5), which gained muscle mass due to myofibre hypertrophy (Fig. 3e). Furthermore, *Prmt5*^*sKO*^ (4.67±2.52, *n*=3) and *Prmt5*^*sKO*^*/mdx* mice (2.80±2.49, *n*=5) displayed a significant reduction of Pax7+ MuSC at 6 months of age compared with controls (Ctrl 15.20±2.39, *n*=5; *mdx* 32.67±18.72, *n*=3; Fig. 3f). We concluded that Prmt5 plays an essential role to maintain the MuSC pool and enables continuous muscle regeneration under chronic disease conditions.

### Prmt5 controls proliferation and differentiation of MuSC

**Figure 4:**
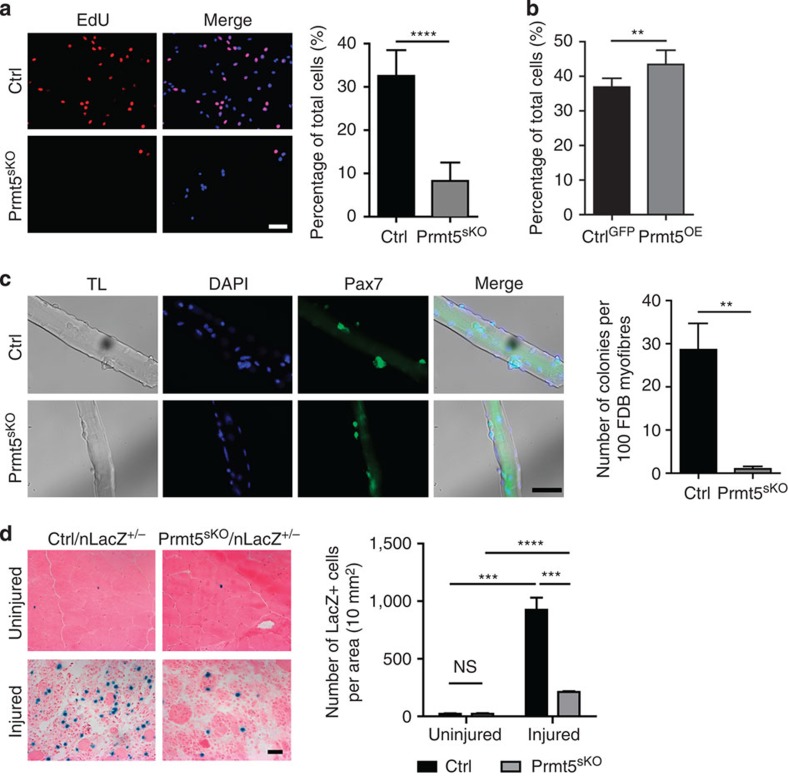
Prmt5 is required for proliferation of MuSC. (**a**) Representative immunofluorescence images of cultured MuSC from control and *Prmt5*^*sKO*^ mice (*n*=6, each) after EdU incorporation. Quantification of EdU^+^ cells is shown on the right. Error bars represent s.d.'s of the mean (*t*-test: ****P<0.0001). (**b**) Percentage of EdU+/MuSC relative to all Pax7^+^ cells after infection with lentiviruses overexpressing Prmt5 (*n*=6) or GFP (*n*=8). Error bars represent s.d.'s of the mean (*t*-test: **P<0.01). (**c**) Immunofluorescence analyses of Pax7^+^-colonies on isolated FDB myofibres of control and *Prmt5*^*sKO*^ mice after 3-day culture (*n*=3, each). Scale bar, 50 μm. Total numbers of Pax7^+^ colonies from 100 myofibres are counted. Quantifications are shown on the right. Error bars represent s.d.'s of the mean (*t*-test: **P<0.01). (**d**) Nuclear LacZ (nLacZ) staining for identification of MuSC in normal and regenerating TA muscle sections from *Prmt5*^*sKO*^ mice and control littermates 3 days after CTX injection (*n*=3, each). Scale bar, 20 μm. The numbers of LacZ^+^ MuSC per 10 mm^2^ section area are displayed. Error bars represent s.d.'s of the mean (*t*-test: ****P<0.0001; ***P<0.001; NS, *P*>0.05; *n*=3). Control (Ctrl): *Pax7CreERT2*^+/−^*/Prmt5*^*+/loxP*^; *Prmt5*^*sKO*^: *Pax7CreERT2*^+/−^*/Prmt5loxP*^*/loxP*^. NS, not significant; TL, transmitted light.

**Figure 5:**
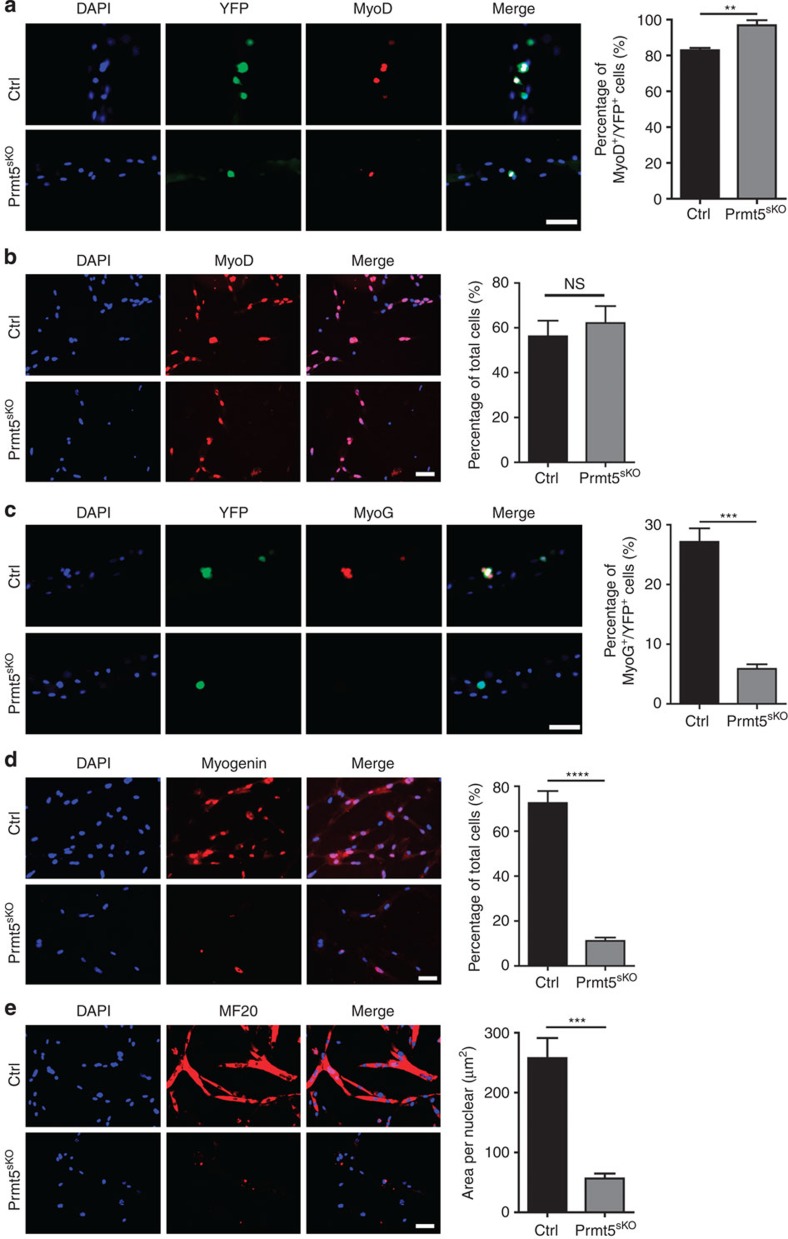
Prmt5 is required for differentiation of MuSC. (**a**,**c**) Immunofluorescence analysis of double YFP^+^/MyoD^+^ cells (**a**) and double YFP^+^ and MyoG^+^ cells (**c**) on isolated FDB myofibres of control and *Prmt5*^*sKO*^ mice after 3-day culture (*n*=3, each). Scale bar, 50 μm. Total numbers of Pax7^+^ colonies from 100 myofibres are counted. Quantifications are shown on the right. Error bars represent s.d.'s of the mean (*t*-test: **P<0.01, ***P<0.001). (**b**) Immunofluorescence staining of FACS-purified YFP^+^ cells cultured for 3 days in proliferation medium for MyoD (*n*=3, each). Scale bar, 50 μm. The percentage of MyoD^+^ cells is shown on the right. Error bars represent s.d.'s of the mean (*t*-test: NS, *P*>0.05). (**d**) Immunofluorescence staining for MyoG of FACS-purified YFP^+^ cells cultured for 3 days in proliferation medium followed by 2 days in differentiation medium (*n*=4, each). Scale bar, 50 μm. The percentage of MyoG^+^ cells is shown on the right. Error bars represent s.d.'s of the mean (*t*-test: ****P<0.0001). (**e**) Immunofluorescence images of MF20^+^ myotubes differentiated from FACS-purified YFP^+^ cells cultured for 3 days in proliferation medium followed by 2 days in differentiation medium (*n*=3, each). Scale bar, 50 μm. The area of MF20^+^ cells is shown on the right. Error bars represent s.d.'s of the mean (*t*-test: ***P<0.001). Control (Ctrl): *Pax7CreERT2*^+/−^*/Prmt5*^*+/loxP*^; *Prmt5*^*sKO*^: *Pax7CreERT2*^+/−^*/Prmt5*^*loxP/loxP*^.

**Figure 6:**
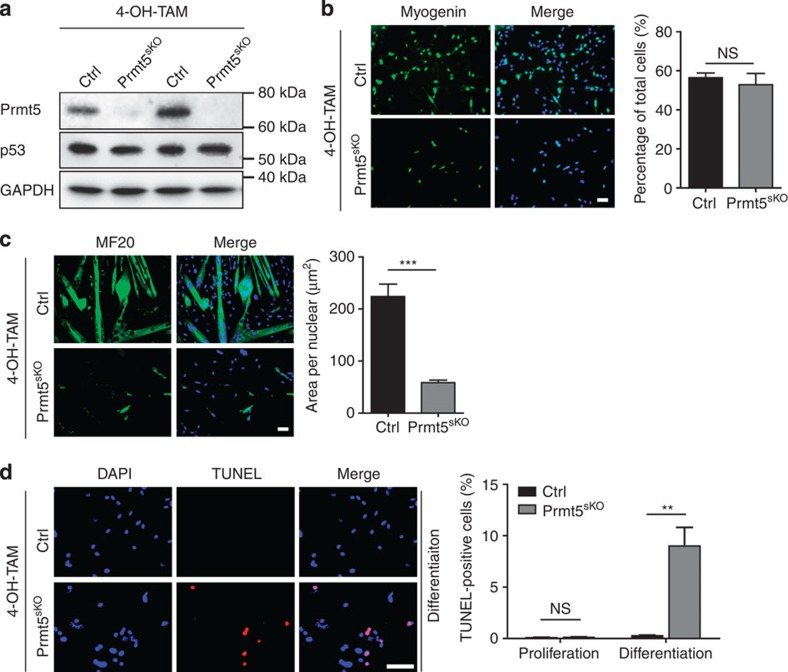
Prmt5 is required for survival of MuSC upon differentiation. (**a**) Western blot analysis of Prmt5 and p53 protein levels in isolated MuSC of *Prmt5*^*sKO*^ and control littermates after *in vitro* 4-OH TAM treatment (*n*=2). (**b**,**c**) Immunofluorescence staining for MyoG (**b**) and MF20 (**c**) of control and *Prmt5*^*sKO*^ MuSC, which were *in vitro* amplified and subsequently treated with 4-OH-TAM, 2 days after induction of differentiation. Scale bars, 20 μm. The percentage of MyoG^+^ and MF20^+^ cells is shown on the right. Error bars represent s.d.'s of the mean. (*t*-test: ***P<0.001; NS, *P*>0.05, *n*=3). (**d**) TUNEL assays of FACS-sorted control and *Prmt5*-deficient MuSC in differentiation medium (*n*=3, each). Scale bar, 20 μm. Quantifications are shown on the right. Error bars represent s.d.'s of the mean (*t*-test: **P<0.01; NS, *P*>0.05). Control (Ctrl): *Pax7CreERT2*^+/−^*/Prmt5*^*+/loxP*^; *Prm5*^*sKO*^: *Pax7CreERT2*^+/−^*/Prmt5*^*loxP/loxP*^. NS, not significant.

To investigate the cellular mechanisms responsible for the loss of the satellite cell pool and impaired muscle regeneration in *Prmt5*^*sKO*^ mice, we first analysed FACS-purified MuSCs from *Prmt5*^*sKO*^ and control mice *in vitro*. *Prmt5*-deficient MuSCs showed a virtually complete arrest of cell proliferation as reflected by a marked reduction of the number of Pax7^+^, 5-ethynyl-2′-deoxyuridine (EdU)-incorporating cells (Ctrl 32.55±5.94%; *Prmt5*^*sKO*^ 8.29±4.24%, each *n*=6), which is in line with the results from Prmt5 knockdown experiments (Fig. 4a). Conversely, lentiviral overexpression of human Prmt5 stimulated proliferation of MuSC indicated by increased EdU incorporation (*Ctrl*^*GFP*^ 36.90±2.52%, *n*=8; *Prmt5*^*OE*^ 43.43±4.13%, *n*=6; Fig. 4b). Moreover, we found a virtually complete absence of the formation of myogenic colonies on single myofibres from FDB muscles of TAM-treated Prmt5^sKO^ mice (1.00±1.00, *n*=3) despite the presence of Pax7^+^ satellite cells (Fig. 4c). Genetic labelling of MuSC using a *Rosa26*^*nlacZ*^ reporter in which removal of a stop-lox cassette by *Pax7*^*CreERT2*^ resulted in activation of *nlacZ* expression uncovered a marked reduction of lacZ-positive MuSC in regenerating muscle of *Prmt5*^*sKO*^ mice 3 days after CTX injection (Ctrl 925±104; *Prmt5*^*sKO*^ 212±6, each *n*=3; Fig. 4d), indicating that Prmt5 is required for MuSC proliferation during the early phase of injury-induced muscle regeneration. Additional lineage tracing of MuSC using a *Rosa26*^*YFP*^ reporter revealed that *Prmt5*-deficient MuSC cells activated *MyoD* expression both on isolated myofibres and in single-cell cultures despite the failure to proliferate, suggesting that activation and proliferation of MuSC are not necessarily linked (Fig. 5a,b). However, activated, non-proliferative *Prmt5*-mutant MuSC failed to differentiate properly. Expression of myogenin (MyoG), an early marker of muscle cell differentiation, was massively reduced in *Prmt5* mutant MuSC attached to myofibres even after extended culture (Ctrl 27.17±2.25%; *Prmt5*^*sKO*^ 5.87±0.78%, each *n*=3; Fig. 5c). In addition, isolated MuSC from *Prmt5*^*sKO*^ mice expressed lower levels of MyoG (Ctrl 72.58±5.30%; *Prmt5*^*sKO*^ 11.13±1.50%, each *n*=4) and did not form MF20^+^ myotubes efficiently (Ctrl 257.50±33.61 μm^2^; *Prmt5*^*sKO*^ 56.53±8.27 μm^2^, each *n*=3; Fig. 5d,e). To further investigate the role of Prmt5 for myogenic differentiation, we inactivated the *Prmt5* gene *in vitro* by treatment with 4-hydroxy-TAM (4-OH) (Fig. 6a) after amplification of isolated *Prmt5*^*sKO*^ MuSC and induction of differentiation. Although we observed expression of the early differentiation marker MyoG in this experimental setting (Fig. 6b), differentiation of MuSC into MF20^+^ myotubes was essentially abrogated (Fig. 6c). We concluded that Prmt5 plays an additional role at a late stage of myogenic differentiation, independent of its function in proliferation and regulation of MyoG expression. Intriguingly, we also detected an increase of apoptosis in isolated *Prmt5*^*sKO*^ MuSCs after induction of differentiation (Ctrl 0.27±0.12%; *Prmt5*^*sKO*^ 9.00±1.75%, each *n*=3), but not under conditions stimulating proliferation (Ctrl 0.08±0.07%; *Prmt5*^*sKO*^ 0.11±0.11%, each *n*=3; Fig. 6d), suggesting that either Prmt5 promotes cell survival during differentiation of MuSC or that lack of proliferation before differentiation favours apoptosis.

### Prmt5 represses the cell cycle inhibitor p21 in MuSC

**Figure 7:**
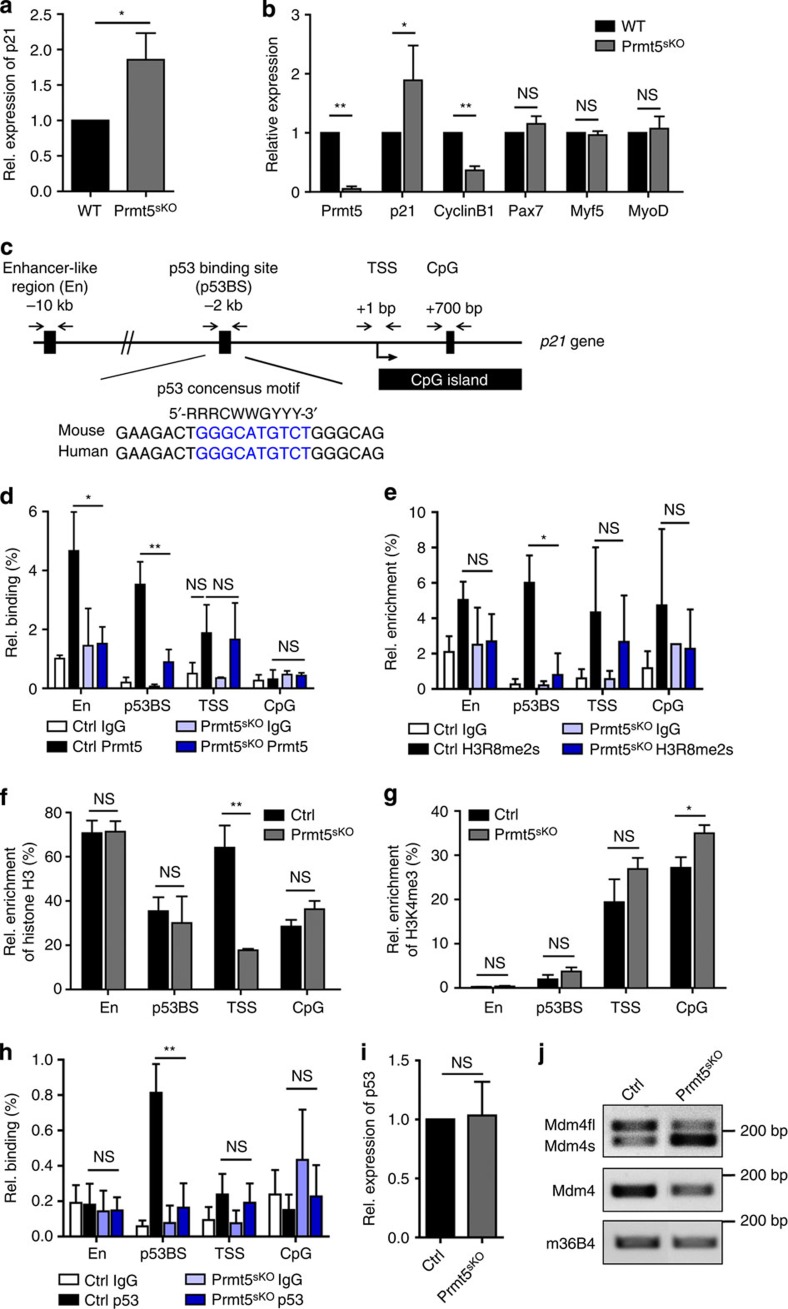
Epigenetic silencing of the cell cycle inhibitor *p21* by Prmt5. (**a**) RT–qPCR analysis of *p21* upregulation in isolated quiescent satellite cells of *Prmt5*^*sKO*^ mice (*n*=4, each). (**b**) RT–qPCR analysis of Prmt5, p21, cyclinB1 and myogenic factors after *Prmt5* inactivation by addition of 4-OH-TAM (*n*=3, each). Expression levels of different mRNAs were normalized to GAPDH mRNA. Error bars indicate s.d. of the mean (*t*-test: *P<0.05; **P<0.01; NS, *P*>0.05). (**c**) Schematic outline of the localization of four important regulatory regions in the *p21* gene locus: enhancer-like (En), p53 binding site (p53BS), transcriptional start site (TSS) and intronic CpG island (CpG). Primer pairs used for ChIP assays are indicated by arrows. The p53 consensus motif of the murine and human *p21* gene locus is shown. (**d–h**) Quantitative PCR analyses of ChIP using antibodies against Prmt5 (**d**), H3R8me2s (**e**), histone H3 (**f**), H3K4me3 (**g**) and p53 (**h**) at indicated regulatory regions of the *p21* gene locus in MuSC of *Prmt5*^*sKO*^ and control mice (*n*=3, each). Relative (Rel.) enrichment of Prmt5, H3 and p53 was normalized to input DNA. Enrichment of H3R8me2 and H3K4me3 was normalized to histone H3. Error bars represent s.d.'s of the mean (*t*-test: **P<0.01; *P<0.05; NS, *P*>0.1). (**i**) RT–qPCR analysis of p53 mRNA levels in MuSC of *Prmt5*^*sKO*^ mice and control littermates (*n*=3, each). Error bars represent s.d.'s of the mean (*t*-test: NS, *P*>0.05). (**j**) Semi-quantitative RT–PCR analysis of different Mdm4 splicing isoforms in MuSC of *Prmt5*^*sKO*^ mice and control littermates. RT–PCR using primers in the 3′untranslated region of Mdm4 detecting both Mdm4fl and Mdm4s was used as loading control. RT–PCR-mediated detection of m36B4 served as an additional loading control. Control (Ctrl): *Pax7CreERT2*^+/−^*/Prmt5*^*+/loxP*^; *Prm5*^*sKO*^: *Pax7CreERT2*^+/−^*/Prmt5*^*loxP/loxP*^. NS, not significant; WT, wild type.

To gain a better mechanistic understanding of the action of Prmt5 in MuSC and to identify genes that might be directly regulated by Prmt5, we performed transcriptome analysis in 4-OH-treated MuSC from control and *Prmt5*^*sKO*^ mice by RNA-sequencing (RNA-seq; Supplementary Data 4). Gene ontology (GO)-term analysis revealed an up- or downregulation of ∼500 genes (false discovery rate <0.05) involved in cell cycle control, DNA metabolism and replication after inactivation of *Prmt5* (Supplementary Fig. 3a,b), which is consistent with the proliferation defects observed in *Prmt5*-deficient MuSC^17^,^21^. The upregulation of the cell cycle inhibitor *p21* attracted our particular attention^22^,^23^,^24^. qRT–PCR analysis of freshly isolated FACS-sorted MuSC confirmed a strong upregulation of *p21* expression in *Prmt5*-deficient MuSCs indicating a transcriptional inhibition of *p21* by Prmt5 (Fig. 7a). In addition, we detected a clear upregulation of *p21* in 4-OH-treated MuSC from *Prmt5*^*sKO*^ mice (Fig. 7b) together with downregulation of *CyclinB1*, a p21 target gene, while transcription of myogenic factors including *Pax7*, *MyoD* and *Myf5* was not altered (Fig. 7b). To investigate a potential direct repression of the *p21* gene by Prmt5, we performed chromatin immunoprecipitation (ChIP) assays concentrating on four well-characterized regulatory regions of the murine *p21* gene: upstream enhancer like region (En), p53 binding site (p53BS), transcriptional start site (TSS) and downstream intronic CpG island (CpG; Fig. 7c; refs 25, 26). In control MuSC, Prmt5 was highly enriched at the En and p53BS but not at the TSS and CpG sites, which was lost after treatment of *Prmt5*^*sKO*^ MuSC with 4-OH (Fig. 7d). Loss of Prmt5 binding caused a significant reduction of H3R8me2s at the p53BS site in *Prmt5*-deficient MuSCs (Fig. 7e), a significant loss of nucleosome occupancy at the TSS site (Fig. 7f) and increased H3K4 trimethylation at the CpG site (Fig. 7g), which is all consistent with suppression of *p21* by Prmt5. Binding of Prmt5 to the p53BS prompted us to ask whether Prmt5 suppresses *p21* expression by preventing recruitment of p53. Surprisingly, inactivation of *Prmt5* prevented binding of p53 to the p53BS in the *p21* locus, thereby suggesting p53-independent upregulation of *p21* in *Prmt5*-deficient MuSCs (Fig. 7h). This conclusion was also supported by normal p53 mRNA and protein levels in *Prmt5* mutant compared with control MuSCs, although we detected accumulation of Mdm4 splicing variants that were shown to stabilize p53 in *Prmt5*-deficient NPCs (Fig. 7i,j; ref. 19).

**Figure 8:**
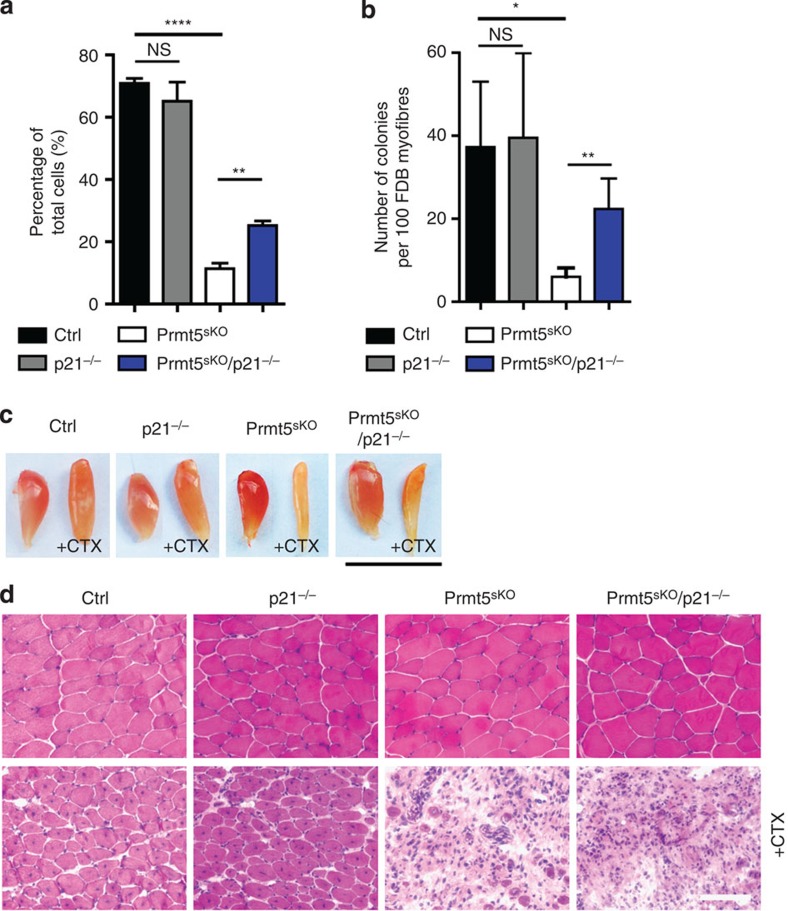
Prmt5 regulates proliferation of MuSC via inhibition of *p21* expression. (**a**) EdU incorporation in isolated MuSCs reveals a partial rescue of MuSC proliferation in *Prmt5*^*sKO*^*/p21*^−/−^ compared with *Prmt5*^*sKO*^ mice (*n*=3, each). Error bars represent s.d.'s of the mean (*t*-test: ****P<0.0001; **P <0.01; NS, *P*>0.05). (**b**) Increased numbers of Pax7^+^ colonies on *Prmt5*^*sKO*^*/p21*^−/−^ compared with *Prmt5*^*sKO*^ 3-day cultured myofibres (*n*=4, each). Numbers of Pax7^+^ colonies from 100 myofibres are shown. Error bars represent s.d.'s of the mean (*t*-test: **P<0.01; *P<0.1; NS, *P*>0.05). (**c**) Representative macroscopic images of non-injured and injured TA muscles of control, *p21*^−/−^, *Prmt5*^*sKO*^ and *Prmt5*^*sKO*^*/p21*^−/−^ mice (*n*=3, each) 14 days after CTX injection. Scale bar, 1 cm. (**d**) Haematoxylin and eosin (H&E) staining of muscle section from non-injured (upper panel) and injured TA (CTX lower panel) muscles of control, *p21*^−/−^, *Prmt5*^*sKO*^ and *Prmt5*^*sKO*^*/p21*^−/−^ mice (*n*=3, each) 14 days after CTX injection. Control (Ctrl): *Pax7CreERT2*^+/−^*/Prmt5*^*+/loxP*^; *Prm5*^*sKO*^: *Pax7CreERT2*^+/−^*/Prmt5*^*loxP/loxP*^. Scale bar, 50 μm.

To investigate whether impaired proliferation of *Prmt5*-mutant MuSC is mediated by p21, we generated *Prmt5*^*sKO*^*/p21*^−/−^ compound mutant mice^22^. Intriguingly, we observed a significant increase of proliferation of MuSC isolated from *Prmt5*^*sKO*^*/p21*^−/−^ (25.25±1.47%, *n*=3) compared with *Prmt5*^*sKO*^ mice (11.34±1.50%, *n*=3; Fig. 8a). Furthermore, we found an increase of the number of myogenic colonies on 3-day cultured FDB myofibres in *Prmt5*^*sKO*^*/p21*^−/−^ mice (22.40±7.30%, *n*=5) compared with *Prmt5*^*sKO*^ mice (6.00±2.16%, *n*=4; Fig. 8b). However, inactivation of *p21* in *Prmt5* mutant mice failed to restore skeletal muscle regeneration, indicating that Prmt5 controls MuSC expansion and differentiation not exclusively by direct regulation of the *p21* gene (Fig. 8c,d).

### Prmt5 is dispensable for embryonic muscle development

**Figure 9:**
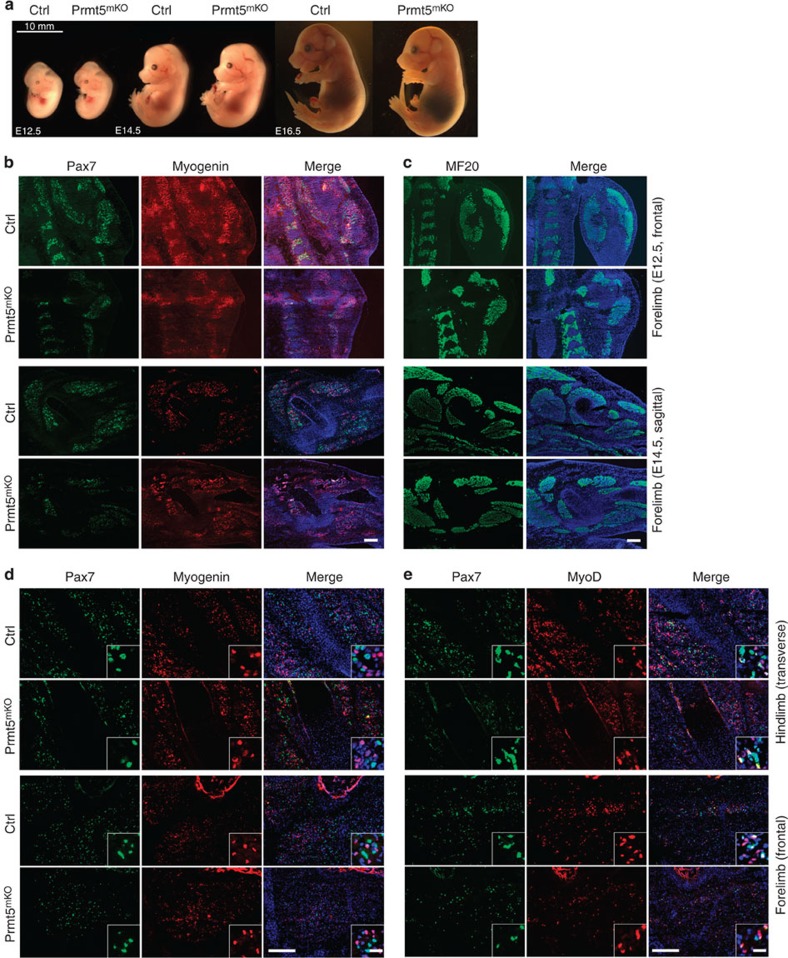
Prmt5 is dispensable in embryonic Pax7^+^ muscle progenitor cells. (**a**) Representative images of control and *Prmt5*^*mKO*^ embryos at embryonic day E12.5, E14.5 and E16.5. (*n*=3, each). Scale bar, 10 mm. (**b**,**c**) Immunofluorescence images of cryosectioned forelimbs and hindlimbs. (**b**) E12.5 frontal sections: upper panel; E14.5 sagittal sections: lower panel, Pax7 (green), myogenin (red), DAPI (blue). Scale bar, 50 μm. (**c**) E12.5 frontal sections: upper panel; E14.5 sagittal sections: lower panel, MF20 (green), DAPI (blue). Scale bar, 50 μm. (**d**,**e**) Immunofluorescence of cryosections from hindlimbs. E16.5 transverse sections: upper panel and forelimbs. E16.5 frontal sections: lower panel, Pax7 (green), myogenin (red; **d**), and Pax7 (green) and MyoD (red; **e**). DNA is stained by DAPI (blue). Scale bars, 50 μm. Scale bars in inserts 20 μm. Control (Ctrl): *Pax7CreERT2*^+/−^*/Prmt5*^*+/loxP*^; *Prm5*^*sKO*^: *Pax7CreERT2*^+/−^*/Prmt5*^*loxP/loxP*^.

It is widely assumed that embryonic and adult myogenesis are regulated by similar molecular cues, although a number of important differences are apparent^10^. Hence, we wanted to know whether Prmt5 does not only control MuSC and muscle regeneration but is also involved in the formation of skeletal muscles during development, in particular, since Prmt5 has been claimed to regulate expression of *Myf5*, *MyoD*, *Myogenin* and *Mef2c* during zebrafish myogenesis^27^. Therefore, we deleted the *Prmt5* gene in the myogenic lineage using the constitutively active *Pax7Cre* knock-in mouse strain (*Pax7*^*Cre*^)^14^. qRT–PCR analysis of FACS-sorted *Pax7*^*ZsGreen*^ myogenic cells from *Pax7*^*Cre*^*/Prmt5*^*loxP/loxP*^ mutant embryos (hereafter referred to as *Prmt5*^*mKO*^) verified efficient inactivation of *Prmt5* expression in embryonic muscle progenitor cells (data not shown). Analysis of control and *Prmt5*^*mKO*^ mutant embryos at E9.5, E12.5 and E16.5 revealed no obvious defects in skeletal muscle formation (Fig. 9a). The normal presence of Pax7^+^, MyoG^+^ and MF20^+^ cells in embryonic forelimbs muscle of E12.5 and E14.5 *Prmt5*^*mKO*^ embryos (Fig. 9b,c) suggested that loss of *Prmt5* in Pax7^+^ myogenic progenitor cells neither affects their expansion nor differentiation during embryonic muscle development. Similarly, lack of *Prmt5* had no effects on Pax7, MyoD and MyoG expression in forelimb and hindlimb muscles at E16.5 when Pax7^+^ muscle progenitor cells play an essential role for fetal muscle growth^28^ or on prenatal muscle growth until birth (Fig. 9d,e). Despite the absence of an apparent skeletal muscle phenotype, most Prmt5^mKO^ mutants died around birth, which we attributed to the activity of Pax7^Cre^ and consecutive loss of *Prmt5* in the central nervous system (CNS)^19^.

## Discussion

Our screen identified several novel potential regulators together with molecules that have already been documented to control the fate of MuSC. Prominent examples include Smad3 (ref. 29) and syndecan-4 (ref. 30). We also identified several epigenetic modifiers including Wdr91, the poly (ADP-ribose) polymerase Parp12 and Ash2l, a component of the Mll2 complex that mediates H3K4 methylation, which has been shown to form a complex with the transcription factor Pax7 to regulate *Myf5* expression and satellite cell proliferation^31^,^32^. The histone arginine methyltransferase Prmt5 attracted our particular attention also because we identified several known interaction partners of Prmt5 in the screen including Myd88 (ref. 33) and Mapk13 (also known as p38delta), a component of the mitogen-activated protein (MAP) kinase pathway^34^, suggesting that Prmt5-dependent mechanisms play a preeminent role in the regulation of MuSC proliferation and differentiation.

**Figure 10:**
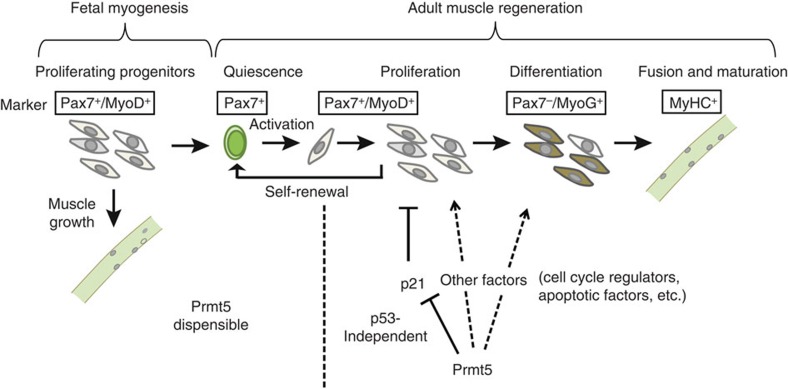
Model of the role of Prmt5 during fetal myogenesis and adult muscle regeneration. Prmt5 controls proliferation of adult MuSC by direct epigenetic silencing of the cell cycle inhibitor *p21* independent of p53, but is dispensable for proliferation and differentiation of Pax7^+^ muscle progenitor cells during fetal myogenesis. Prmt5 does not affect the initial activation of MuSC resulting in MyoD expression but enables proliferation of MuSC, thus generating a poised state, which keeps MuSC in a standby mode allowing rapid MuSC amplification under disease conditions. Inactivation of *Prmt5* in proliferating MuSC does not suppress MyoG expression but blocks differentiation, thereby suggesting an additional function of Prmt5 for muscle cell differentiation. Proliferating fetal muscle progenitor cells are defined by expression of Pax7 and MyoD, quiescent MuSC by expression of Pax7 and lack of MyoD expression, proliferating MuSC by the concomitant expression of Pax7 and MyoD, and differentiating muscle cells by expression of MyoG. Differentiated muscle cells are marked by expression of myosin heavy chain (MyHC).

During adulthood MuSC mostly exist in a resting, quiescent state but must be able to expand rapidly in order to regenerate damaged muscle tissue. Relaxed control of quiescence might lead to over-proliferation, depletion of the stem cell pool and might favour tumour formation. Failure to respond appropriately to proliferative cues will impair self-renewal of MuSC and compromise regeneration. Prmt5 seems to be a decisive component of the regulatory network that maintains this intricate balance and keeps MuSC in a poised standby mode (Fig. 10). In contrast, embryonic myogenesis is characterized by the rapid expansion of myogenic progenitor cells, which need to form skeletal muscles in a relatively short time period alleviating the need to enter a quiescent, non-proliferative state. Hence, it makes sense that the role of Prmt5 in the regulation of cell proliferation differs significantly between embryonic and adult myogenesis, whereas the control of muscle lineage determination and differentiation seems to follow a similar pattern^10^,^35^,^36^,^37^.

A major function of Prmt5 for conferring a reversible resting state to MuSC is apparently the restriction of *p21* expression. Reduced expression of *Prmt5* in MuSC will result in upregulation of *p21*, which increases the threshold for cell cycle re-entry (Fig. 10). Although we detected Prmt5 by immunofluorescence in virtually all MuSC, its level of activity and hence regulation of *p21* might vary, thereby contributing to the heterogeneity of MuSC. MuSC with lower Prmt5 activity might constitute a reserve population that is only activated under severe stress conditions. Alternatively, differential regulation of Prmt5 activity in asymmetrically dividing MuSC might distinguish cells returning to quiescence from those that undergo rapid expansion. Careful quantitative evaluation of Prmt5 activity in single MuSC will solve these questions in the future.

We do not claim that the epigenetic repression of *p21* is the only mechanism by which Prmt5 arrests MuSC proliferation, in particular, since RNA-seq analysis identified several additional cell cycle regulators that might also be regulated by Prmt5 either directly or indirectly. Inactivation of *p21* in *Prmt5*-deficient MuSC failed to rescue muscle regeneration fully, although proliferation of MuSC could be partially restored, indicating different modes of action of Prmt5 independent of p21. In fact, additional functions of Prmt5 in the regulation of progenitor cell behaviour have been reported previously. In embryonic stem (ES) cells, Prmt5 promotes pluripotency by modulating the cytoplasmic LIF/Stat3 signalling pathway, indirectly suppressing genes that are associated with ES cell differentiation^17^. In NPCs, Prmt5 regulates alternative splicing of Mdm4 that in turn stabilizes p53, which causes upregulation of p21 and inhibition of cell cycle progression^19^. Superficially, the findings in NPC appear to partially recapitulate the situation in MuSC, but a more careful analysis reveals fundamental differences in the mode of action. Inactivation of *Prmt5* in MuSC does not change the mRNA and protein level of p53, although alternative splicing of Mdm4 was altered. Furthermore, we found that binding of p53 to the *p21* locus was lost after inactivation of *Prmt5*, indicating that activation of *p21* in *Prmt5*-deficient MuSC does not depend on p53.

Although suppression of MuSC expansion by upregulation of *p21* dominated the phenotype of *Prmt5*^*sKO*^ mice, lineage-tracing experiments revealed that *Prmt5*-deficient MuSC failed to express myogenin, indicating that *Prmt5* mutant MuSCs are unable to differentiate and form myofibres *in vivo*. This conclusion was also supported by the failure of MuSC to form myotubes even when *Prmt5* was deleted after initiation of *MyoD* expression, a phenomenon that was also observed in C2C12 myoblasts^38^. In addition, the timed inactivation of *Prmt5* in differentiating MuSC suggests an additional role for terminal myogenic differentiation after expression of MyoG has commenced. Interestingly, induction of differentiation of *Prmt5* mutant MuSC triggered apoptosis, which might be related to the differentiation block and contribute to the loss of MuSC during regeneration and ageing.

Our study revealed that inactivation of *Prmt5* in MuSC of *mdx* mice resulted in a severe loss of muscle volume and recapitulated several symptoms of human Duchenne muscular dystrophy within 90 days. The findings emphasize the pivotal role of MuSC in maintaining muscle mass under disease conditions. A similar phenotype was described recently using *mdx* mice completely lacking telomerase activity (*mdx/mTR*^2G^ mice)^20^. However, in *mdx/mTR*^2G^ mice, a massive atrophy of the diaphragm was only visible after 60 weeks, indicating that the lack of *Prmt5* had more severe consequences in MuSC function than loss of telomerase activity. We believe that *Prmt5*^*sKO*^*/mdx* mice might serve as a valuable model to study effects of therapeutic interventions on dystrophin-deficient myofibres without the interference of MuSC constantly replenishing lost or damaged myofibres.

Remarkably, muscle mass remained rather stable in *Prmt5*^*sKO*^ mice under physiological conditions for at least 3 months despite a significant decline of the number of MuSC and the failure of MuSC to expand. This finding allows two conclusions: (i) under physiological conditions, MuSC contribute only to a minor degree to the maintenance of muscle mass; and (ii) a significant proportion of MuSC undergoes self-renewal during a 3-month period. However, the second conclusion has to be viewed with caution, since it is possible that the lack of Prmt5 induces cell death of MuSC without prior activation and induction of proliferation, although we did not find evidence for such a scenario in our experiments. In the future, it will be interesting to further exploit the *Prmt5*^*sKO*^ model (Fig. 10) to study the role of MuSC in muscle dystrophies, analyse their function in muscle hypertrophy or to block proliferation of tumour cells in rhabdomyosarcomas.

## Methods

### Animals

The *Prmt5*^*loxP/loxP*^ mouse strain was obtained from EUCOMM. Rosa26^nlacZ^ and *C57BL/10ScSn-Dmdmdx/J* (*mdx*) mouse strain was obtained from The Jackson Laboratory (Bar Harbor, ME). Generation of *Pax7*^*CreERT2*^ (ref. 7), *Rosa26*^*YFP*^ (ref. 39) *Pax7*^*ICN*^ (ref. 14), *p21* null (ref. 22), *Pax7*^*ZsGreen*^ (ref. 12) and *Z/EG* reporter^6^ mice have been described previously. Primers used for genotyping are shown Supplementary Table 1. TAM (Sigma) was administered intraperitoneally at 3 mg per 40 g body weight per injection. CTX (0.06 mg ml^−1^, Sigma) was injected into TA muscles in a volume of 50 μl. All animal experiments were done in accordance with the Guide for the Care and Use of Laboratory Animals published by the US National Institutes of Health (NIH Publication No. 85-23, revised 1996) and according to the regulations issued by the Committee for Animal Rights Protection of the State of Hessen (Regierungspraesidium Darmstadt).

### Myofibre isolation and MuSC purification

The FDB muscles were isolated and digested with 0.2% collagenase P (Roche) in DMEM medium. The isolated myofibres were either fixed directly with 4% paraformaldehyde (PFA) or fixed after 3-day culturing in DMEM medium with 20% fetal calf serum (FCS) and basic fibroblast growth factor (bFGF) (5 ng ml^−1^). Satellite cell isolation and purification were performed according to established methods^6^. Briefly limb and trunk muscles were minced, digested with 100 CU Dispase (BD) and 0.2% type II collagenase (Worthington Biochemicals), and consecutively filtered through 100-, 70- and 40-μm cell strainers (BD). Cells were applied to a discontinuous Percoll gradient consisting of 70% Percoll overlayed with 30% (vol/vol) Percoll. Mononuclear cells were collected at the 70/30 interphase and subjected to FACS (BD FACSAriaII) either using immunostaining with fluorescence-coupled primary antibodies (CD11b^−^, CD45^−^, CD31^−^, CXCR4^+^ and CD34^+^;refs 6,13,40) or GFP fluorescence of satellite cells from *Pax7*^*ZsGreen*^ mice and *Pax7*^*CreERT2*^*/Rosa26*^*YFP*^ mice. FACS-purified SCs were cultured on Matrigel-coated 384-well μClear plates (BD Biosciences, Greiner) in DMEM medium with 20% FCS and bFGF (5 ng ml^−1^;refs 6,8). EdU incorporation assay was performed by adding EdU with a final concentration of 10 μM 3 h before fixation and then analysed using the Click-iT EdU kit (Invitrogen) according to the manufacturer's protocol. TUNEL assays to monitor apoptosis were carried out with the In Situ Cell Death Detection Kit (Roche) according to the manufacturer's protocol. For Cre recombinase-mediated *in vitro* ablation of Prmt5, cultured satellite cells were treated with 4-OH-TAM (0.4 mM, Calbiochem) for 4 days and were analysed 5 days later. Lentiviruses overexpressing the coding region of human *Prmt5* were generated with a modified lentiviral vector derived from plko.1 (Sigma-Aldrich) in HEK293T cells using the helper plasmids pMD2.G and psPAX2, and used for infection of MuSC.

### Immunofluorescence and morphological analysis

Cultured cells and myofibres were fixed in 4% PFA. Frozen muscle sections (5–10 μm) were fixed in cold acetone. Primary antibodies for immunohistochemical staining are shown in Supplementary Table 2. LacZ staining of regenerating or normal TA muscle sections 3 days after CTX injection was carried out according to standard protocols, Eosin staining was used to visualize muscle fibres. Masson's Trichrome staining was carried out using the ACCUSTAIN trichrome staining kit (Sigma-Aldrich) following the manufacturer's instructions.

### ChIP and RT–qPCR

ChIP assays on purified satellite cells were performed according to established protocols modified for small cell numbers^41^. FACS-purified satellite cells (100,000) were first crosslinked with 1% formaldehyde for 10 min and then quenched by 0.125 M glycine for 15 min at room temperature. Chromatin was sheared to lengths of 300–500 bp using Bioruptor (Diagenode) and subjected to immunoprecipitation with antibodies are shown in Supplementary Table 2. Primers used for ChIP–qPCR are shown in Supplementary Table 3. For RT–qPCR assays, total RNA from satellite cells and muscles was isolated using Trizol reagent (Invitrogen) according to the manufacturer's protocol. An amount of 1 μg of purified RNA was subjected to reverse transcriptase reaction in the presence of 25 ng ml^−1^ random primers and 2.5 mM dA/C/G/TTP with 10 U ml^−1^ SuperScript II Reverse Transcriptase (Invitrogen). Primers used for RT–qPCR in Supplementary Table 4.

### Western blot analysis

For western blot assays *in vitro* cultured satellite cells were harvested, washed with ice-cold PBS and lysed in cell lysis buffer (20 mM Tris (pH 7.5), 400 mM NaCl, 1 mM EDTA, 1 mM EGTA, 1% Triton X-100, 2.5 mM sodium pyrophosphate, 1 mM β-glycerophosphate, 1 mM Na_3_VO_4_ and 1 μg ml^−1^ leupeptin). Whole-cell lysates (10 μg) were subjected to SDS–PAGE and western blotting using antibodies are shown in Supplementary Table 2. Protein expression was visualized using an enhanced chemiluminescence detection system (GE Healthcare, Little Chalfont, UK) and quantified using a ChemiDoc gel documentation system (Bio-Rad). Uncropped images of original western blots are presented in (Supplementary Fig. 4).

### RNA-seq

For RNA-seq, RNA was isolated from cultured satellite cells using the miRNeasy micro Kit (Qiagen) combined with on-column DNase digestion (DNase-Free DNase Set, Qiagen) to avoid contamination by genomic DNA. RNA and library preparation integrity were verified with a BioAnalyzer (Agilent). Ribosomal depletion was performed using RiboMinus Eukaryote System v2 (Life Technologies) with 500 ng total RNA as input following the low input protocol. Libraries were prepared with the Ion Total RNA-Seq Kit v2 (Life Technologies) adjusting the total amount of depleted and fragmented RNA to standard protocols. Sequencing was performed on the Ion Torrent Proton platform using V3 chemistry (Ion PI Template OT2 200 Kit v3, Life Technologies) and PIV2 Chips (Ion PI Chip Kit v2, Life Technologies). For each run, two RNA-seq libraries were measured resulting in 82M reads in total and at least 40M reads per library. Raw reads were assessed for quality, adaptor content and duplication rates with FastQC 0.10.1, trimmed by Reaper version 13–100 (ref. 42) and terminally aligned to the Ensemble mouse genome version mm10 (GRCm38) by STAR 2.4.0a (ref. 43). Differentially expressed genes were identified using DESeq2 version 1.62 (ref. 44). Only genes with a minimum fold change of ±2, a maximum Benjamini–Hochberg corrected *P* value of 0.05 and a minimum combined mean of 5 reads were classified as significantly differentially expressed. The Ensemble annotation was enriched with UniProt data (release 06.06.2014) based on Ensemble gene identifiers. Gene ontology analysis was performed using DAVID Bioinformatics Resources (http://david.abcc.ncifcrf.gov)^45^. Heatmaps of cell cycle-related genes were generated using ‘heatmap.2' in R ‘gplots' package. Gene expression data were deposited in the NCBI Geo database (http://www.ncbi.nlm.nih.gov/geo/query/acc.cgi?acc=GSE66822) under the accession number GSE66822.

### Mass spectrometry

Muscle and satellite cell samples were homogenized in lysis buffer (4% SDS in 100 mm Tris/HCl, pH 7.6). Lysates were loaded on an SDS–PAGE (NuPAGE 4–12% BisTris gel, Invitrogen) and stained with colloidal Protein Staining Solution (Invitrogen). Evenly sized gel pieces were excised for in-gel digestion using trypsin after reduction and alkylation of gel pieces. Gel pieces were washed twice with 50% (50 mM NH_4_HCO_3_/50% ethanol) for 20 min, dehydrated with 100% ethanol for 10 min and vacuum dried. Gel pieces were reduced with 10 mM dithiothreitol for 45 min at 56 °C and alkylated with 55 mM iodocetamide (BioUltra, Sigma-Aldrich) for 30 min at room temperature in the dark. After two steps of washing/dehydration, samples were dehydrated twice with 100% ethanol for 15 min and vacuum dried. Gel pieces were digested overnight at 37 °C in 50 μl of digestion buffer containing 12.5 ng μl of Sequencing Grade Modified Trypsin (Promega Corp., Madison, USA). Released peptides were extracted (collecting separately the liquid mixture of each samples at each step) once by adding 100 μl of 30% acetonitrile liquid chromatography/mass spectrometry (LC/MS) grade (Thermo Scientific)/3% trifluoroacetic acid (Sigma-Aldrich) in water, and twice by adding 70% acetonitrile, followed by two final extractions with 100% acetonitrile. Extracts were vacuum dried to remove acetonitrile and subsequently acidified with 0.5% trifluoroacetic acid (TFA)^46^. Peptides were purified by stop and go extraction tips^47^, and were run on a LC system coupled to a LTQ-Orbitrap XL or a LTQ-Orbitrap Velos mass spectrometer (Thermo Fisher Scientific) equipped with a nanoelectrospray source (Proxeon). Full-survey scan spectra (*m/z*=300–1,650) were acquired in the Orbitrap with a resolution of *R*=60,000 after accumulation of 1,000,000 ions. Raw data were analysed using the MaxQuant software package^48^. Database searches were performed with the Mascot search engine against a murine FASTA database (IPI 3.54). A false discovery rate of 1% was used, and only peptides with a minimum of six amino-acid length with at least one unique peptide were included for data analysis.

### High-throughput screen and hit validation

A customized shRNA library against selected genes was robotically re-arrayed from a murine genome-wide The RNAi Consortium (TRC) shRNA library^49^ (Sigma-Aldrich). Plasmids were purified with GenElute HP 96-Well Miniprep Kits (Sigma-Aldrich) and tested for integrity with a PvuII digest. High-throughput production of lentivirus was performed by Ca_3_(PO_4_)_2_ transfection of HEK293T cells with helper plasmids pMD2.G and psPAX2. Four-hundred fifty freshly isolated satellite cells per well were seeded into 384-well tissue culture plates freshly coated with Matrigel (Greiner). Twenty-four hours later, cells were transduced with lentiviral supernatants supplemented with 8 μg ml^−1^ polybrene for 6 h. After media exchange, cells were incubated for 72 h. Each 384-well plate contained 56 controls including 12 individual GFP-producing lentiviruses to monitor transduction efficiency, four positive controls for Pax7 knockdown (shRNA Pax7) and four positive controls for Nf1 knockdown (shRNA Nf1). Cells were fixed in 4% PFA and whole-well images were acquired and analysed using an ImageXpress Micro automated high-throughput fluorescence microscope and MetaXpress software (Molecular Devices). Pax7/DAPI ratios were determined for each individual well. Only plates with *Z*′ values >0.5 according to 1−(2*σ*_pos_ +2*σ*_neg_/*μ*_neg_−*μ*_pos_) were used for further processing. Values as reference for Pax7 expression in percentage were then calculated according to *z*=(*x*/*μ*) (−1) (*x*, value of particular sample; *μ*, mean of plko1 empty vector; *n*=4). Target genes qualified as hits if percentiles were higher or lower than 25% compared with control.

### Magnetic resonance imaging

All MRI experiments were performed on a 7.0-T superconducting magnet (Bruker Biospin, Pharmascan, 70/16, 16 cm; Ettlingen, Germany) equipped with an actively shielded imaging gradient field of 300 mT m^−1^ (ref. 50). The frequency for the ^1^H isotope is 300.33 MHz. A 60-mm inner diameter linear-polarized ^1^H volume resonator was used for RF pulse transmission and signal reception (Bruker Biospin). Localized images were acquired using a spin-echo sequence and corrections of slice angulation were performed, if necessary. RARE (Rapid Acquisition with Relaxation Enhancement) sequences (repetition time (TR)=2,500 ms, echo time (TE)=36.7 ms, slice thickness=1 mm) in axial and coronal orientation were used to determine exact positioning of the lower part of the mouse body. A coronal MSME (Multi-Slice-Multi-Echo)-spin-echo-sequence with an echo time TE=8.6 ms, repetition time TR=453 ms, a field of view FOV=7 × 7 cm^2^, matrix size MTX=512 × 256 and a slice thickness of 1 mm was recorded. Volumetric quantification of fat and muscle tissue from images was processed by software ImageJ. A list of anatomically defined landmarks was used to derive tissue-specific signal intensity thresholds and to define the region of interest for intensity sensitive region growing segmentation. The resulting tissue voxel volumes inside the region of interest were determined as cubic millimetres for each tissue class. Mice were measured under volatile isoflurane (1.5–2.0% in oxygen and air with a flow rate of 1.0 l min^−1^) anaesthesia; the body temperature was maintained at 37 °C by a thermostatically regulated water flow system during the entire imaging protocol.

### Statistics

For statistical analysis, the two following tests (two-tailed) were used: (1) three or more groups: one factorial ANOVA; (2) two groups: unpaired *t*-test. *P* values <0.05 were considered statistically significant. Data were analysed using GraphPad Prism v5.03 (GraphPad Software, San Diego, CA).

## Additional information

Accession number in NCBI Geo database (http://www.ncbi.nlm.nih.gov/geo): GSE66822.

**How to cite this article**: Zhang, T. *et al.* Prmt5 is a regulator of muscle stem cell expansion in adult mice. *Nat. Commun.* 6:7140 doi: 10.1038/ncomms8140 (2015).

## Supplementary Material

Supplementary Information Supplementary Figures 1-4 and Supplementary Tables 1-4

Supplementary Data 1 Results of Mass Spectrometry analysis for different cell populations from skeletal muscle. Tab I: Raw data of all identified proteins from all samples including the proteomes of purified myotubes, non-purified satellite cells, FACS purified (GFP+) and (GFP-) cells. Tab II: Raw data of the FACS purified (GFP+) SC Proteome. Tab III: Unique peptides that were exclusively identified in FACS purified (GFP+) SC Proteome. Tab IV: MuSC-specific proteome. All proteins identified in FACS purified (GFP-) proteome were subtracted from the FACS purified (GFP+) proteome. Remaining peptides were sorted based on peptide intensities (Column Q). Remaining peptides that were identified in FACS purified (GFP+) MuSC proteome were kept if intensities were higher than in the myotube proteome. The list corresponds to all initially targeted genes depending on availability of shRNAs. Tab V: MuSC-specific proteome. All proteins identified in FACS purified (GFP-) proteome were subtracted from the FACS purified (GFP+) proteome. Remaining peptides were sorted based on the sum of all peptides (Column K). Remaining peptides that were identified in FACS purified (GFP+) MuSC proteome were kept if intensities were higher than in the myotube proteome. Tab VI: Identical to Tab V but intensities of myotube proteome (Column T) were subtracted. Tab VII: Identical to Tab VI but sorted on values of Column T (Intensities of peptides from FACS-sorted cells). Tab VIII: Identical to Tab VI but sorted on values of Column F (Presence of peptides from FACS-sorted cells).

Supplementary Data 2 List of all genes that were targeted in the functional screen

Supplementary Data 3 Results of the functional shRNA screen against the satellite cell proteome. Tab 1: Pax7/DAPI scores normalized to empty vector control. Tab 2: Target mRNAs with scores higher and lower of 25th percentile with two independent shRNAs. Tab 3: Target mRNAs with scores higher of 25th percentile with two independent shRNAs: Pax7 UP. Tab 4: Target mRNAs with scores lower of 25th percentile with two independent shRNAs: Pax7 DOWN. Tab 5a: Overlap of hits with opposing phenotypes. Tab 5b: Scores of overlap of hits with opposing phenotypes. Tab 6: Target mRNAs with scores higher of 25th percentile with two independent shRNAs and opposing phenotypes subtracted. Tab 7: Target mRNAs with scores lower of 25th percentile with two independent shRNAs and opposing phenotypes subtracted. Tab 8: Complete target list. 7 mRNAs that were targeted were scored as artifacts after manual reevaluation of images.

Supplementary Data 4 Results from RNA-seq of Prmt5sKO vs. Wild-type MuSC. Tab1: Overview of sample runs showing statistics of raw reads, trimming, mapping performance and number of significantly differentially regulated genes according to DESeq2. Spearman correlations of replicates are included. Tab2: Differential regulation tests according to DESeq2 filtered for significance (FC > 2, sum (counts) > 2, FDR < 0.05).
