# Supplementary material for: Prmt5 is a regulator of muscle stem cell expansion in adult mice
Source: Nat Commun. 2015 Jun 1;6:7140. doi: 10.1038/ncomms8140 (PMC4458870; doi:10.1038/ncomms8140)
Supplement: Supplementary Information — Supplementary Figures 1-4 and Supplementary Tables 1-4 [file ncomms8140-s1.pdf]

Supplementary Information

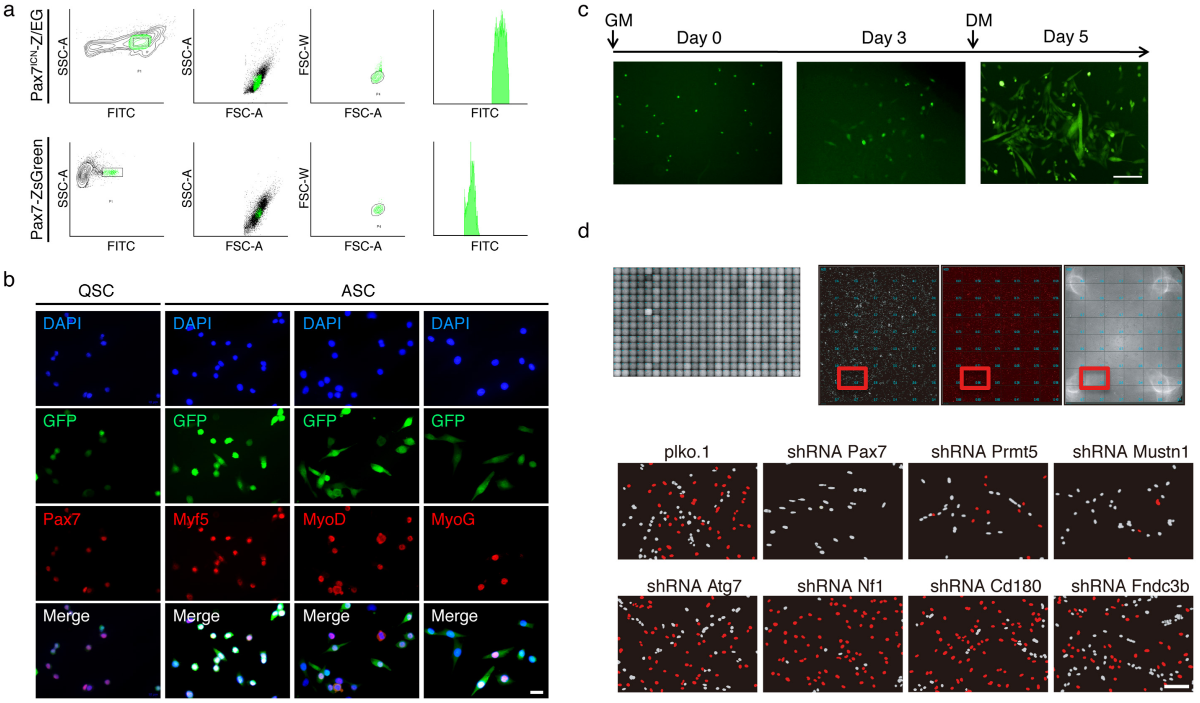

Supplementary Figure 1: Identification of new regulators of MuSC by a proteome-based shRNA screen. (a) FACS plots of GFP$^+$ and GFP$^-$ cells from *Pax7^{ICN}-Z/EG* (upper panel) and *Pax7^{ZsGreen}* (lower panel) reporter mice**. (b)** Immunofluorescence staining of myogenic factors in freshly isolated, quiescent (QSC) and 3 day cultured activated (ASC) satellite cells from *Pax7^{ICN}-Z/EG* mice. **(c)** Expansion and differentiation of FACS purified MuSC from *Pax7^{ICN}-Z/EG* mice *in vitro*. Scale bar 100 µm. **(d)** Upper panel: Images of a 384-well plate used for HTS. Lower panel: representative images of candidate target mRNAs that promoted (Nf1, Cd180 and Fndc3b), or inhibited expansion of MuSC (Pax7, Prmt5 and Mustn1) or had no effects (Atg7) after shRNA-mediated knockdown compared to control vectors (plko.1). Scale bar 50 µm.

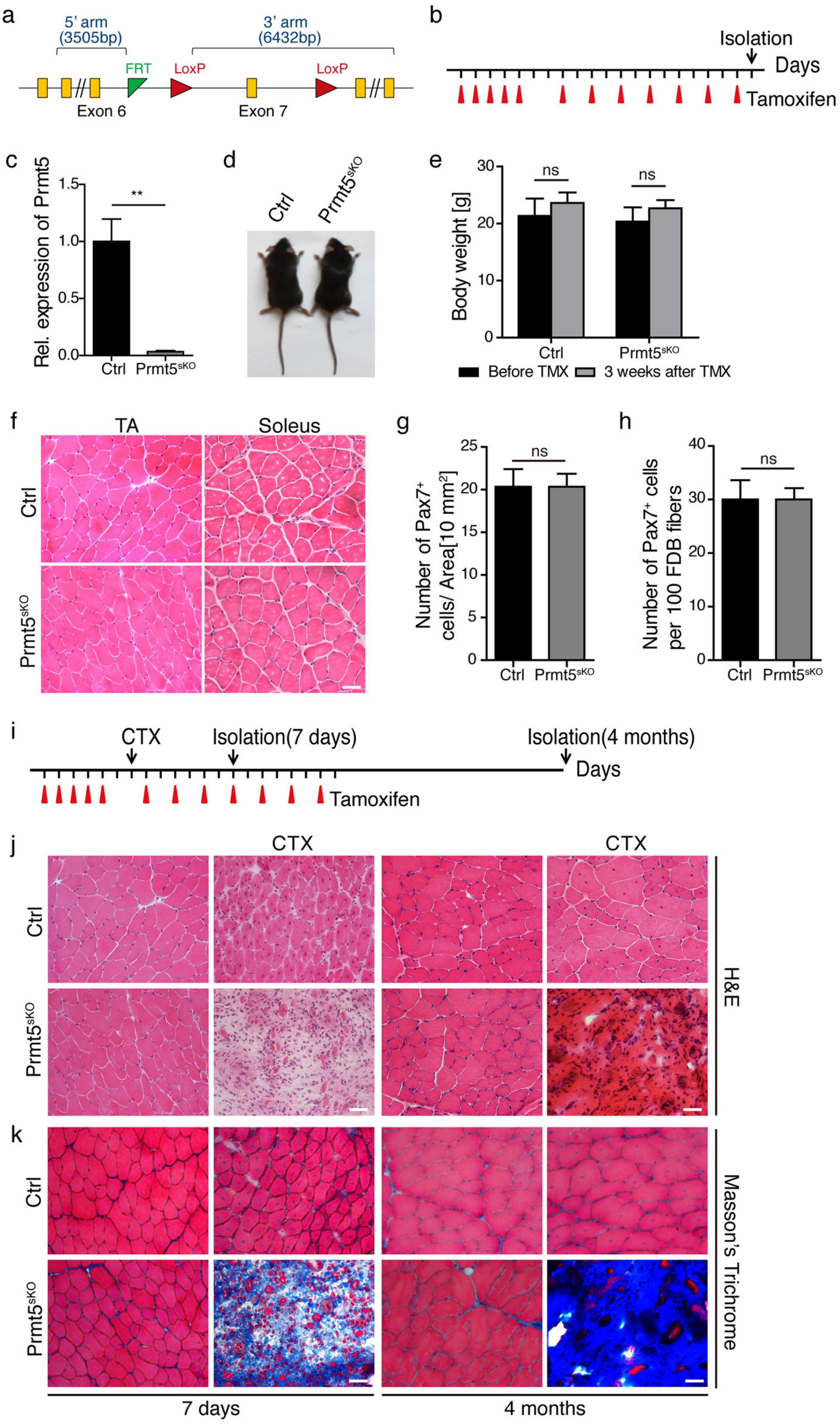

Supplementary Figure 2

Supplementary Figure 2: Prmt5 is dispensable for short-term maintenance of MuSC and skeletal muscle integrity. **(a)** Schematic outline of the *Prmt5* gene locus with the floxed exon 7 that can be deleted by Cre recombinase-mediated recombination. Red arrowheads indicate positions of loxP sites. **(b)** Schematic outline of the TAM administration regimen. **(c)** RT-qPCR analysis of Prmt5 in isolated quiescent satellite cells of Ctrl (n=3) and *Prmt5*^{sKO} (n=4) mice. Error bars represent standard deviations of the mean (t-test: **p < 0.01). **(d)** Representative images of control and *Prmt5*^{sKO} mice after TAM administration for 21 days. **(e)** Bodyweight of control and *Prmt5*^{sKO} mice after administration of TAM for 3 weeks. (n=3, each). Error bars represent standard deviations of the mean (t-test: ns p>0.5, n=3). **(f)** H&E staining of TA and Soleus muscles after TAM treatment of control and *Prmt5*^{sKO} mice (n=3, each). Scale bar 20 µm. **(g, h)** Numbers of Pax7 expressing cells (Pax7^{+}) on TA cryosections (g) or freshly isolated FDB myofibers (h) from control and *Prmt5*^{sKO} mice 3 weeks after TAM treatment obtained by immunofluorescence staining (n=3, each). The numbers of Pax7^{+} cells per 10 mm^2 section area (g) or 100 myofibers (h) are displayed. Error bars represent standard deviations of the mean (t-test: ns p>0.5). **(i)** Schematic outline of the TAM regimen and CTX injection used for analysis of short-term (7 days) and long-term (4 months) muscle regeneration in control and *Prmt5*^{sKO} littermates (n=3, each). Muscle regeneration was analyzed at different time points as indicated. **(j, k)** H&E (j) and Masson's Trichrome staining (k) of injured or non-injured TA muscles 7 days or 4 months after CTX injection showing impaired muscle regeneration and fibrosis of injured muscle in *Prmt5*^{sKO} mice compared to control littermates (n=3, each). Scale bar 20 µm.

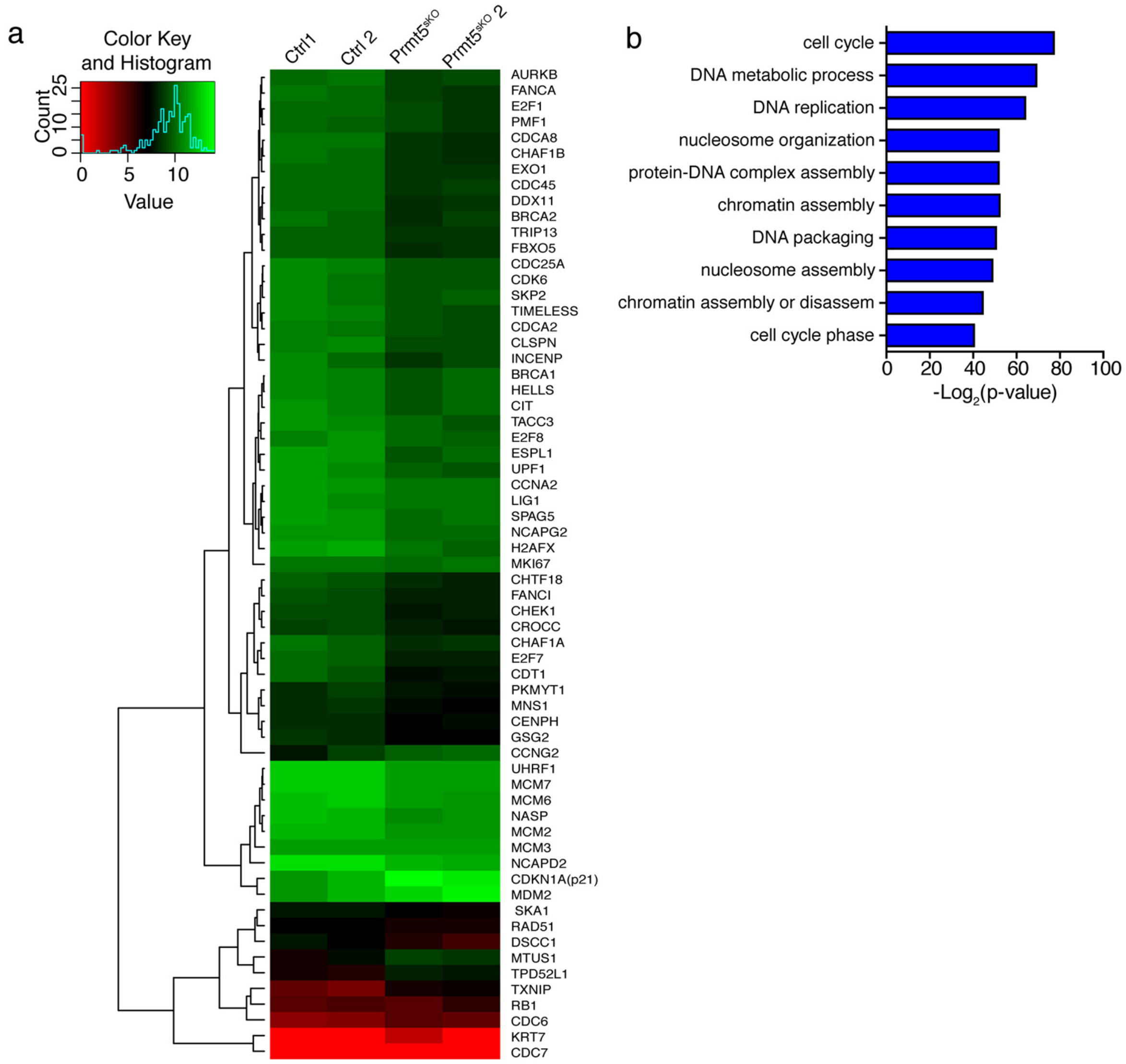

Supplementary Figure 3: RNA-seq and GO-analysis of up- and down-regulated genes in *Prmt5* deficient MuSC. (**a**) Heat map of selected up- and down-regulated genes after inactivation of *Prmt5* in MuSC (n=2, each). (**b**) GO term analysis of biological processes that are affected by the loss of *Prmt5* in MuSCs (n=2, each).

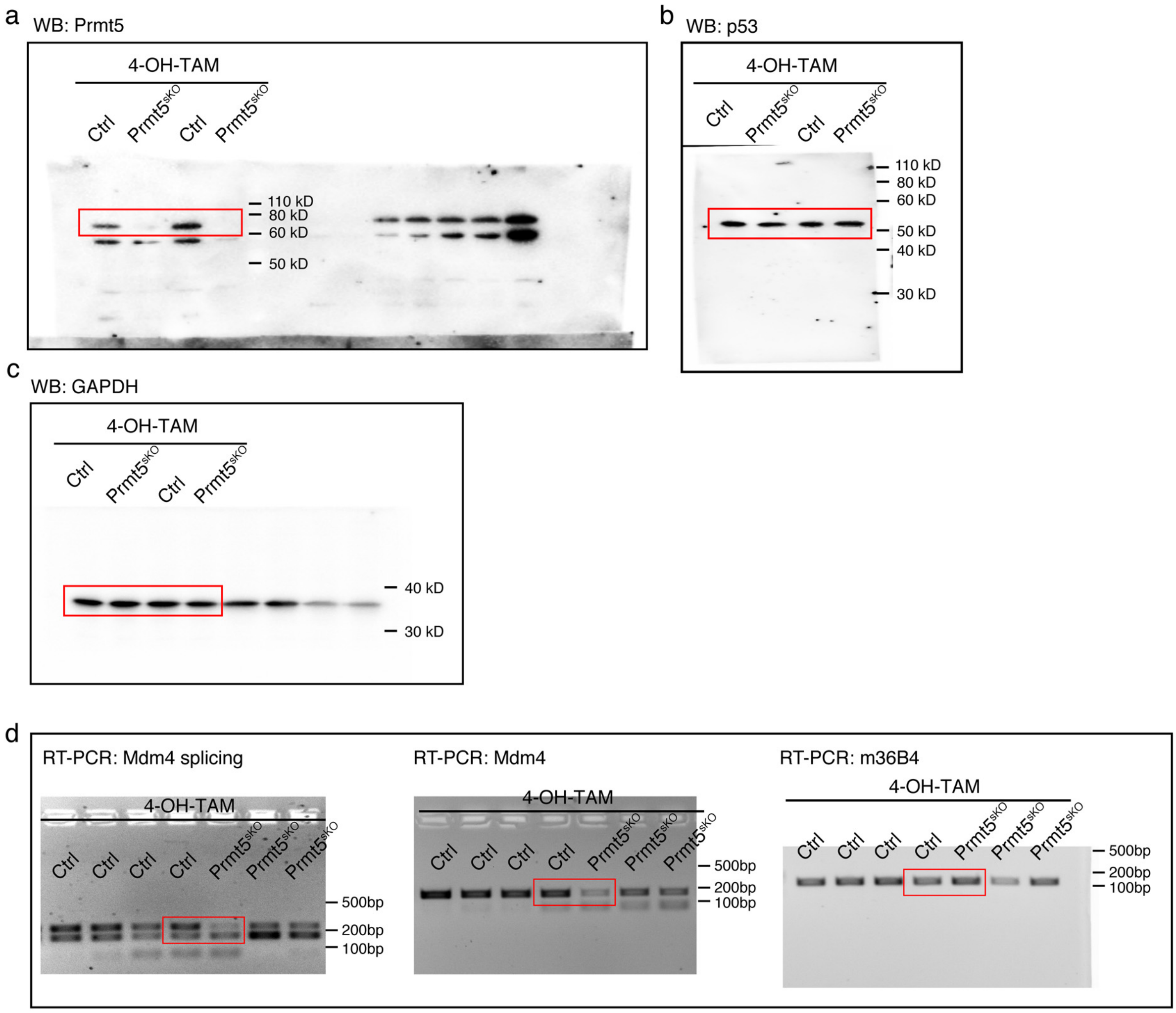

Supplementary Figure 4: (**a, b, c**) Uncropped blots for Figure 6. **(d)** Uncropped gels for Figure 7.

Genotyping	Primer	Sequence (5'-->3')
Pax7^{CreERT2}	Forward	ACTAGGCTCCACTCTGTCCTTC
	Reverse	GCAGATGTAGGGACATTCCAGTG
ZsGreen	Forward	CTGCATGTACCACGAGTCCA
	Reverse	GTCAGCTGCCACTTCTGGTT
Rosa26^{YFP}	RosaFA	AAAGTCGCTCTGAGTTGTTAT
	RosaRF	GGAGCGGGAGAAATGGATATG
	Rosa-SpliAC	CATCAAGGAAACCCTGGACTACTG
Mdx	mdx_Forward	GCGCGAAACTCATCAAATATGCGTGTTAGTGT
	mdx WT Reverse	GATACGCTGCTTTAATGCCTTTAGTCACTCAGATAGTTG AAGCCATTTTG
	mdx MT Reverse	CGGCCTGTCACTCAGATAGTTGAAGCCATTTTA
p21	p21 exon 144	GAACTTTGACTTCGTCACGG
	p21 genoU	ACAACACCTCCTGGTCAGAGG
	p21 PGK neo3	GAAGAACGAGATCAGCAG
Pax7$^{ICN-Cre}$	ck188	GCTCTGGATACACCTGAGTCT
	ck256	TCGGCCTTCTTCTAGGTTCTGCTC
	ba97	GATCTGGACGAAGAGCATCA
	ck172	GGATAGTGAAACAGGGGCAA
Prmt5	Loxp3 Forward	CCAGAACTTCCTCTGGTTTCTGG
	LoxP3 Reverse	GAAAGCTGTGTGTCCTCACAC

Supplementary Table 1: List of primers used for genotyping of different mouse strains.

**Antibody**	**Antigen**	**Application**	**Working Concentration**	**Manufacturer**
**Anti-Pax7 mouse**	Pax7	IF	0.5 µg/ml	DSHB
**Anti-Myf5 rabbit**	Myf5	IF	0.2 µg/ml	Santa Cruz
**Anti-MyoD rabbit**	MyoD	IF	1 µg/ml	Santa Cruz
**Anti-MyoD1 mouse**	MyoD	IF	0.5 µg/ml	LSBio
**Anti-Myogenin rabbit**	Myogenin	IF	0.2 µg/ml	Santa Cruz
**Anti-Myogenin mouse**	Myogenin	IF	1:20 dilution	DSHB
**MF20**	Myosin heavy chain	IF	1:20 dilution	DSHB
**Anti-Gapdh**	Gapdh	WB	1:2000 dilution	Cell signaling
**Anti-Prmt5**	Prmt5	WB,ChIP	0.3 µg/ml, 1 µg/100 µl	Active Motif
**Anti-p53**	p53	WB,ChIP	1:1000, 1:100 dilution	Cell signaling
**Anti-H3R8me2s**	H3R8me2s	ChIP	1 ug/100 µl	Novus
**Anti-Histone H3**	Histone H3	ChIP	1 ug/100 µl	Abcam
**Rabbit IgG**		ChIP	1 ug/100 µl	Diagenode
**Mouse IgG**		ChIP	1 ug/100 µl	Diagenode
**Anti-CD11b PE-Cy7**	CD11b	FACS	2 µg/ml	eBioscience
**Anti-CD45 PE**	CD45	FACS	2 µg/ml	eBioscience
**Anti-CD31 PE**	PECAM1	FACS	2 µg/ml	eBioscience
**Anti-CXCR4 APC**	CXCR4	FACS	2 µg/ml	eBioscience
**Anti-CD34 A450**	CD34	FACS	2 µg/ml	eBioscience
**Anti-YFP**	YFP	IF	0.2µg/ml	Evrogen

Supplementary Table 2: List of antibodies. Antibodies were used for immunofluorescence (IF), western blotting (WB), chromatin immunoprecipitation (ChIP), and FACS analysis (FACS).

ChIP-qPCR	Primer forward (5’-3’)	Primer reverse (5’-3’)
**p21 enhancer**	CACAGGGAAGAGAGCTCCAG	AGCCAGGGCTACACAGAGAA
**p21 TSS**	TCCACAGCGATATCCAGACA	GGACACACCTGTGACTCTGG
**p21 p53 binding site**	CAAGCCCTTCCCAGACTTCC	TCTAGAGATCGCTGCCCAGA
**p21 CpG island**	CCTGTTTCGCGGTAGCCA	ACAATGAGTCACCTCCTCGC

Supplementary Table 3: List of primers used for ChIP-qPCR experiments.

RT-qPCR	Primer forward(5'-3')	Primer reverse(5'-3')
**Prmt5 Exon5-7**	AGAATGCCCCGACTACACAC	AGCCTCTGCTGCACCTTAGA
**p21**	CGGTGTCAGAGTCTAGGGGA	ATTGGAGTCAGGCGCAGATC
**p53**	TGCTGTGCAATTAAAGGCTGT	CGTGTTCTCCGAGATACTTGGT
**Pax7**	GCTACCAGTACAGCCAGTATG	GTCACTAAGCATGGGTAGATG
**Myf5**	CCACCTCCAACTGCTCTGAC	GCTTCAGGGCTTCTTTTCCT
**MyoD**	GAATGGCTACGACACCGCCTACTAC	CCTACGGTGGTGCGCCCTCTGC
**Myogenin**	TTGCTCAGCTCCCTCAACCA	TGGGCTGGGTGTTAGTCTTA
**m36B4**	AGATTCGGGATATGCTGTTGGC	TCGGGTCCTAGACCAGTGTTC
**Mdm4**	AGTCAGGTGCGGCCAAAA	CCCAAAAGATCTCCACCACA
**Mdm4 splicing**	TGTGGTGGAGATCTTTTGGG	TCAGTTCTTTTTCTGGGATTGG

Supplementary Table 4: List of primers used for RT-qPCR analysis.
